# Mycoplasma infection mimicking a malignancy in a waldenstrom macroglobulinemia patient

**DOI:** 10.1186/s12879-023-08163-6

**Published:** 2023-04-07

**Authors:** Junqing Wu, Donghua He, Fang Yu, Yue Huang, Meiru Bian, Chengxuan Yu, Jiao Liu, Zhen Cai, Yi Zhao

**Affiliations:** 1 Bone Marrow Transplantation Center, The First Affiliated Hospital, Zhejiang University School of Medicine, Hangzhou, 310000 China; 2 Liangzhu Laboratory, Zhejiang University Medical Center, Hangzhou, 310000 China; 3 Department of Pathology, The First Affiliated Hospital, Zhejiang University School of Medicine, Hangzhou, 310000 China; 4 Department of Hematology, The Second People’s Hospital of Huai’an, The Affiliated Huai’an Hospital of Xuzhou Medical University, Huaian, 223001 China; 5 Department of Colorectal Surgery and Oncology, Key Laboratory of Cancer Prevention and Intervention, The Second Affiliated Hospital, Ministry of Education, Zhejiang University School of Medicine, Hangzhou, 310000 Zhejiang China; 6 Hangzhou Xixi Hospital, Zhejiang University of Traditional Chinese Medicine, Hangzhou, 310000 Zhejiang China; 7 Institute of Hematology, Zhejiang University, Hangzhou, 310000 Zhejiang China; 8 Zhejiang Laboratory for Systems & Precision Medicine, Zhejiang University Medical Center, Hangzhou, 310000 China

**Keywords:** Mycoplasma hominis, Waldenstrom macroglobulinemia, ^18^F-FDG-PET/CT, Bladder cancer

## Abstract

**Background:**

Mycoplasma hominis infection is common in urinary tract. ^18^F-FDG-PET/CT is a valuable tool for tumor and infection diagnosis. Few studies have shown the ^18^F-FDG-PET/CT images after mycoplasma infection.

**Case presentation:**

Here we described a case of Waldenstrom macroglobulinemia with thickened bladder wall. The ^18^F-FDG-PET/CT showed the SUVmax up to 36.1 mimicking bladder cancer. The results of histopathological examination and metagenomic sequencing of the blood and urinary revealed the Mycoplasma hominis infection.

**Conclusion:**

The full consideration should be given to the possibility of infection besides tumor in lesions with high SUV value in ^18^F-FDG-PET/CT, especially in immunodeficiency patients.

**Supplementary Information:**

The online version contains supplementary material available at 10.1186/s12879-023-08163-6.

## Introduction

Waldenstrom macroglobulinemia (WM) is a rare lymphoma. It accounts for approximately 1% of non-Hodgkin lymphoma [1]. Due to immune deficiency, patients are more susceptible to infection. Mycoplasma hominis is the colonizer of the urogenital tract. It is associated with chronic urogenital infections [2].

The ^18^F-FDG-PET/CT is the nuclear imaging technique for diagnosing infectious diseases and tumors. Both of the inflammatory and malignant cells have high glucose metabolism, and cause elevated FDG uptake [3]. However, few studies have shown the ^18^F-FDG-PET/CT images after mycoplasma infection.

Here we described the case of a patient with WM, who had a high fever. The ^18^F-FDG-PET/CT showed the thickening of the bladder wall with the SUVmax up to 36.1, which led us to mistake it for a bladder cancer. However, the histopathology examination showed the infiltration of inflammatory cell, and the results of metagenomic sequencing of blood and urinary both showed the infection of Mycoplasma hominis.

## Case description

A 59-year old male had high fever around 38–40℃ for more than 10 days. In local hospital, the hematological parameters were as follows, WBC 3.88 × 10^9^/L, NEU 85.3%, Hb 77 g/L, PLT 157 × 10^9^/L, CRP 92.05 mg/L. He was treated with cefoperazone/sulbactam sodium, imipenem/cilastatin sodium and linezolid for 5 days. However, his symptoms were not relieved. He had a history of WM for more than 1 year, and received 6 courses of Bendamostine plus Rituximab regimen.

Then he came to our hospital, and the hematological parameters here were as follows, WBC 4.3 × 10^9^/L, NEU 80.7%, Hb 86 g/L, PLT 221 × 10^9^/L, CRP 57.4 mg/L, PCT 0.25ng/mL, IgA 19 mg/dl, IgG 258 mg/dl, IgM 1673 mg/dl. The result of blood culture was negative. No RNA of influenza A and B, parainfluenza I, II and III, and respiratory syncytial virus was detected in sputum. No DNA of respiratory adenovirus and mycoplasma pneumoniae was detected in sputum.

**Fig. 1:**
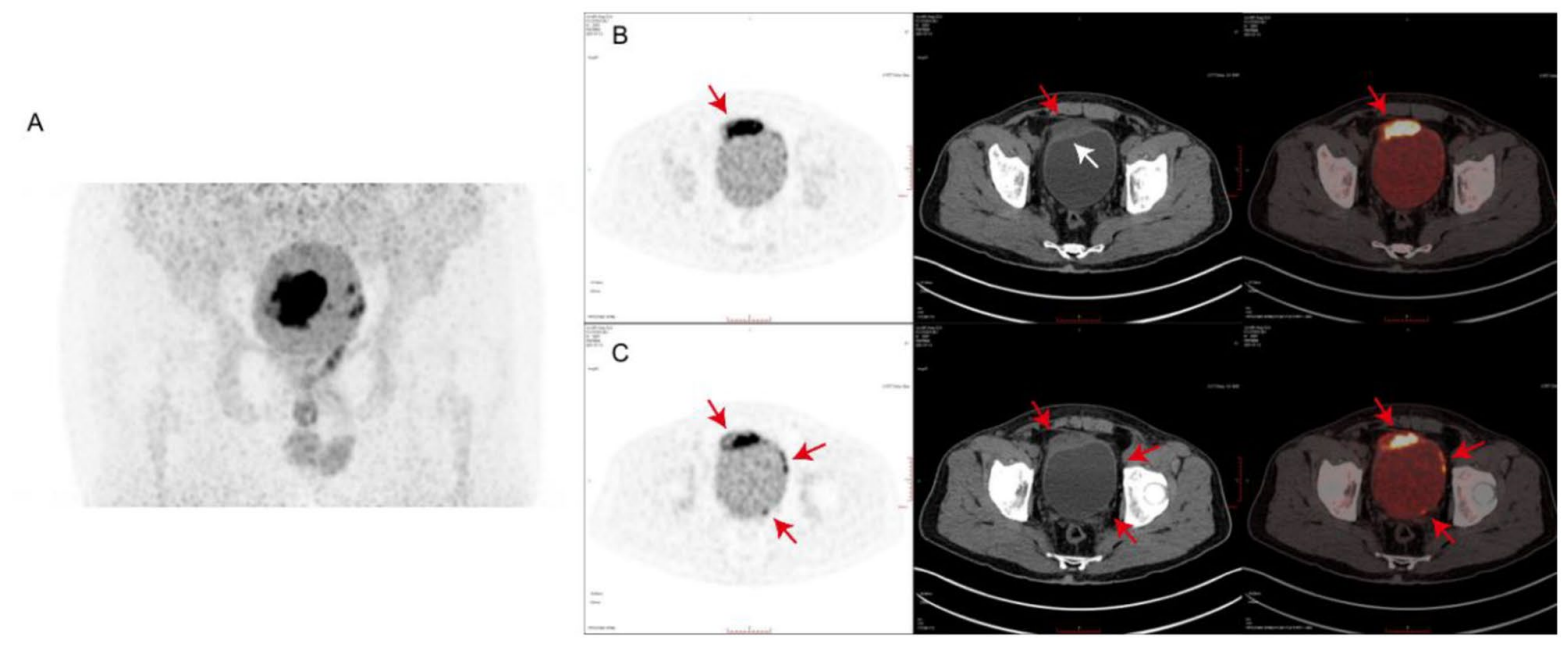
The FDG uptake of the bladder (A), the anterior wall(B), the left anterior and the left posterior (C) wall in 18 F-FDG-PET/CT. The SUVmax was 36.1 in the anterior wall. The SUVmax was 11.5 in the left anterior wall and the left posterior wall. The red arrows show the FDG accumulation sites. The white arrow shows the biopsy site

**Fig. 2:**
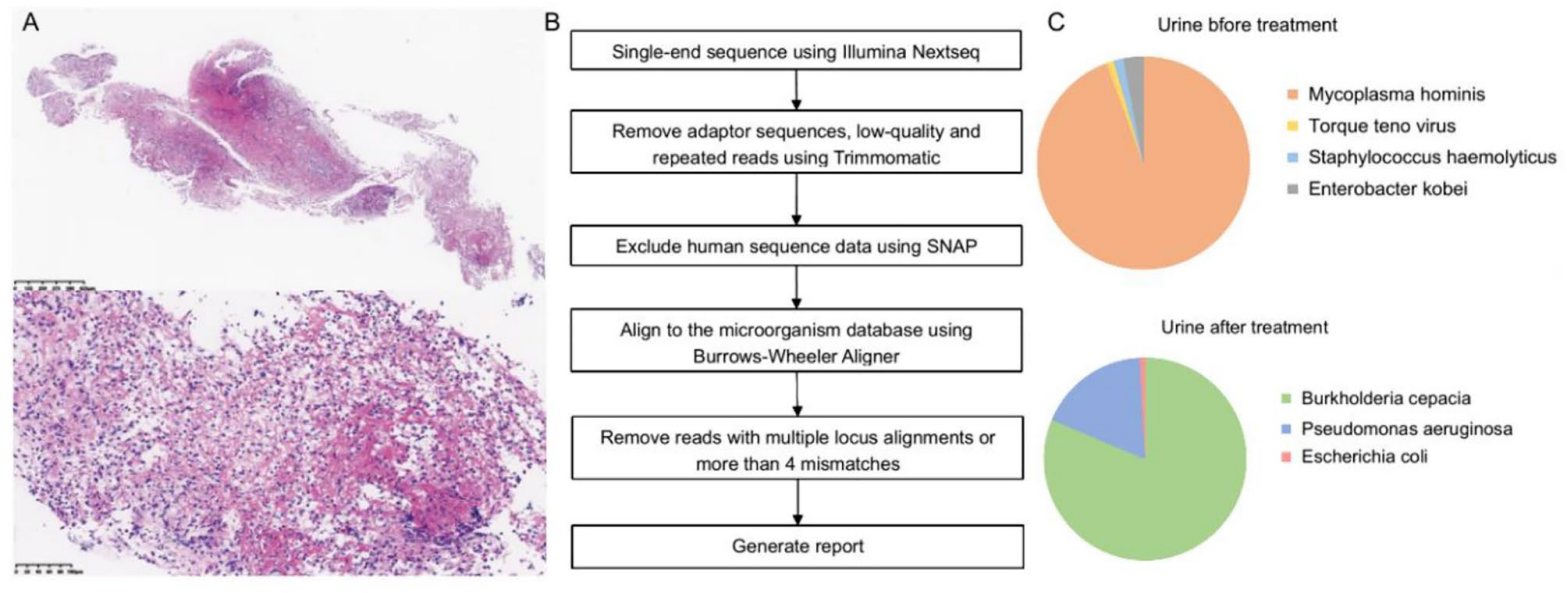
The histopathology of the infiltration of inflammatory cells (A). The bioinformatics pipeline of the metagenomic sequence (B). The abundances of the taxa in urine before and after treatment (C)

Although no pathogen was found in blood and sputum, and he had no symptoms of frequency and urgency of micturition, we noticed that the urine routine test showed WBC 843/µl and bacteria 155.8/µl. We then performed the urine culture test and the ultrasound examination of bladder. The result of the urine culture was negative. The ultrasound examination showed the thickening of the anterior wall of the bladder, with a range of about 4.2 × 1.4 cm. The thicken wall partially protruded outside the bladder, with a range of about 1.5 × 1.1 cm. To assess the progress of WM and the lesions in the bladder, we performed ^18^F-FDG-PET/CT. Figure 1A showed the frontal image of bladder, and there was a large accumulation of FDG. The SUVmax was up to 36.1 in the anterior wall (Fig. 1B). In addition, the left anterior wall and the left posterior wall of the bladder were slightly thickened. The SUVmax was 11.5 (Fig. 1C). The red arrows in Fig. 1 show the FDG accumulation sites. With a suspicion of malignancy, histopathological examination was performed after cystoscopy. The white arrow in Fig. 1B shows the biopsy site. The result turned out to be the infiltration of inflammatory cell (Fig. 2A). Since the expression of CD20, CD19, Kappa and Lambda were negative, we believed there were no WM cells (Supplementary Fig. 1A). Moreover, we used shotgun metagenomics for metagenomic sequencing. The bioinformatics pipeline is showed in Fig. 2B, and the results of blood and urinary both showed the Mycoplasma hominis infection (Fig. 2C and Supplementary Fig. 1B). After using tigecycline and piperacillin/tazobactam sodium, his temperature was finally under control.

One year later, he came to our hospital for recheck. The ultrasound examination showed no thickening of the anterior wall of the bladder. The cystoscopy showed that some mucous membranes were rough, but no new organism was found. The metagenomic sequencing of urinary showed no Mycoplasma hominis infection (Fig. 2C).

## Discussion

WM is characterized by lymphoplasmacytic infiltration of the bone marrow and the presence of IgM monoclonal protein. Clinical features include anemia, thrombocytopenia, hepatosplenomegaly, lymphadenopathy, and hyperviscosity [4]. For our patient, the increased IgM protein was 57.1 g/L, and the decreased hemoglobin was 116 g/L at diagnosis. The bone marrow routine test showed abnormal lymphocytes accounting for 39%, mature plasma cells accounting for 8%, and immature plasma cells accounting for 2%. The bone marrow immunophenotyping showed abnormal lymphocytes accounting for 37%. The pathological results of bone marrow showed the infiltration of abnormal small lymphoid cells. There were 2.7% of MYD88 L265P mutated cells in bone marrow. Therefore, the WM diagnosis was established.

WM patients have an increased risk of infections [5]. Because the polyclonal immunoglobulins in WM patients decreased, leading to the defective antibody response [6]. It has been reported that patients with monoclonal gammopathy, including WM, had a twofold increased risk of developing infection [6]. HCV infected people have a threefold increased risk of WM [6, 7].

Mycoplasma hominis is an important pathogen in human genitourinary tract. In men with normal immune system, the mycoplasma infection is usually asymptomatic and most men resolve infection without developing disease [8]. When the patient has infection symptoms, but the standard culture is negative and there is no reaction to routine antibiotics, mycoplasma should be actively searched [9]. The risk factors of mycoplasma infection in genitourinary tract include increased numbers of sex partners, unsafe sexual practices, lack of education and migrant background [8, 10]. The patient in our case was an elderly man who used to be a PE teacher and had a harmonious family. It seems that there were no risk factors mentioned above. From the perspective of mechanism, the mycoplasma attaches to the host epithelial cells and rapidly invades them. The host immune cells activate defense pathways and secrete proinflammatory cytokines to resist the infection [11]. We believe that the decrease of polyclonal immunoglobulin in WM patients results in the deficiency of immune response, which causes the symptoms. Besides, the mycoplasma infection is significantly associated with HIV infection, and the mechanism may also involve immune deficiency [12].

Mycoplasma has a potential role in cell malignant transformation and chromosomal instability. Mycoplasma infection is observed in the circulating tumor cells of patients with carcinoma, and it promotes the migratory capacity of malignant cells [9]. Mycoplasma hominis is one of the most detected mycoplasma from cancer patients [13]. However, there is no report on mycoplasma infection in WM.

Besides the Mycoplasma hominis, we detected other bacteria in urine and blood. In urine, Torque teno virus, Staphylococcus haemolyticus and Enterobacter kobei were detected when the patient first came to our hospital. However, among all sequences, the sequences of Mycoplasma hominis accounted for 94.40%. Burkholderia cepacia, Pseudomonas aeruginosa and Escherichia coli were detected when he came to our hospital for recheck. Due to the control of WM, these bacteria did not cause fever. Mycoplasma hominis was in high abundance in urine at initial stage, but disappears after treatment. In blood, we detected Mycoplasma hominis, Torque teno virus, Human betaherpesvirus 5, Staphylococcus haemolyticus, Propionibacterium acnes and Moraxella osloensis. Torque teno virus is a part of the normal mammalian virome [14]. Human betaherpesvirus 5 is also called Cytomegalovirus (CMV). No IgG or IgM of CMV were detected in blood. Staphylococcus haemolyticus, Propionibacterium acnes and Moraxella osloensis are normal microorganisms of human skin [15, 16]. Tigecycline is a kind of tetracycline, which is effective against Mycoplasma hominis [17]. After using tigecycline and piperacillin/tazobactam sodium, his temperature was under control. Therefore, we believed that Mycoplasma hominis was the pathogen causing fever in this patient.

The ^18^F-FDG-PET/CT is an important method for the evaluation of patients with fever of unknown origin [18]. It combines high spatial resolution with detection of increased glycolysis [19]. Although the CT, MRI, and ultrasound are also able to detect the infectious processes, the substantial anatomical changes are absent in the early phase, and the infectious lesions remain undetected. It has the advantages of higher resolution, higher sensitivity in chronic low-grade infections [20]. However, the imaging of mycoplasma infection in ^18^F-FDG-PET/CT has rarely been reported.

Meanwhile, the ^18^F-FDG PET/CT is valuable for evaluation of tumor [21]. Unlike the other urologic malignancies such as prostate cancer and renal cell carcinoma, bladder cancer is highly avid for glucose, which is essential for the employment of ^18^F-FDG PET/CT. However, the physiologic FDG activity excretes through the urinary system. The FDG activity in urine interfere with tumor activity, which may mask FDG accumulation of bladder cancer. Thus hindering the detection and local staging in bladder cancer [22]. To overcome this limitation, profuse water uptake, use of diuretics, and voiding with catheter should be taken into consideration [23]. The ^18^F-FDG PET/CT is also an important tool in rendering decisions regarding radiotherapy, chemotherapy, and post-operative follow-up [24]. It has been reported that the higher SUVmax was correlated with higher recurrence risk independent of pathological findings after 2 years of follow-up [25]. The SUVmax above 6 predicts a poorer outcome [26].

Since the FDG uptake might correlate with both the inflammation and the malignancy, the pathological biopsy is still the gold standard in diagnosis [22]. Besides, the metagenomic sequencing allows sequencing of the pathogen’s genome directly from the clinical specimens, which is useful in investigating the pathogenic microorganisms [2].

## Conclusion

We first showed the ^18^F-FDG-PET/CT images of Mycoplasma hominis infection in bladder in a patient with WM. The ^18^F-FDG-PET/CT is a valuable tool for detecting tumor. However, full consideration should be given to the infection in lesions with high SUV, especially in immunodeficiency patients. The pathological biopsy is the gold standard and metagenomic sequencing is an important assistant.

## Electronic supplementary material

Below is the link to the electronic supplementary material.

Supplementary Material 1 Figure 1

## Data Availability

All the data and material involved in the current study are available from the corresponding author on reasonable request.

## Abbreviations

WM: Waldenstrom macroglobulinemia
WBC: White blood cell
NEU: Neutrophil
Hb: Hemoglobin
PLT: Platelet
CRP: C-reactive protein
PCT: Procalcitonin
