# Supplementary material for: Mycoplasma infection mimicking a malignancy in a waldenstrom macroglobulinemia patient
Source: BMC Infect Dis. 2023 Apr 7;23:219. doi: 10.1186/s12879-023-08163-6 (PMC10080790; doi:10.1186/s12879-023-08163-6)
Supplement: Supplementary file 1 — Supplementary Material 1 Figure 1 [file 12879_2023_8163_MOESM1_ESM.docx]

Supplementary figure 1


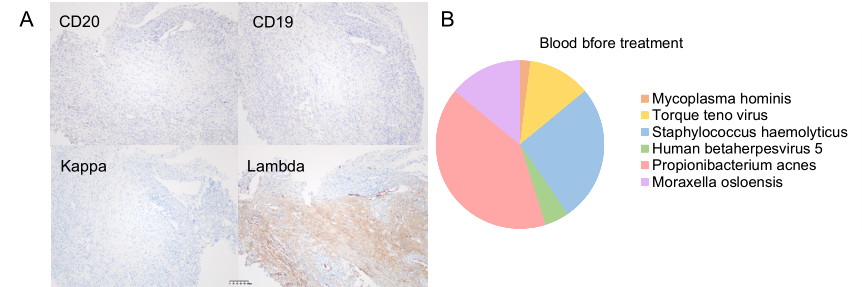


The histopathology of CD20, CD19, Kappa and Lambda (A). The abundances of the taxa in blood before the treatment (B).
